# Culture and Quality Matter in Building Effective Mentorship Relationships with Native STEM Scholars

**DOI:** 10.1093/biosci/biac064

**Published:** 2022-08-03

**Authors:** Mica Estrada, Gerald Young, Lilibeth Flores, Paul R Hernandez, K Kanoho Hosoda, Kathy DeerInWater

**Affiliations:** Institute for Health and Aging, University of California, San Francisco, in San Francisco, California, United States; University of California, Berkeley, Berkeley, California, United States; Institute for Health and Aging, University of California, San Francisco, in San Francisco, California, United States; Department of Teaching, Learning, and Culture, College of Education and Human Development, Texas A&M University, College Station, Texas, United States; Institute for Health and Aging, University of California, San Francisco, in San Francisco, California, United States; American Indian Science and Engineering Society, Albuquerque, New Mexico, United States

**Keywords:** STEM, mentorship, Native Americans, American Indian, higher education

## Abstract

Native peoples (Native American, Alaskan Native, Native Hawaiian) are underrepresented in academia; they represent 2% of the US population but 0.01% of enrolled undergraduate students. Native peoples share the experiences of colonization and forced assimilation, resulting in the loss of ancestral knowledge, language, and cultural identity. Recognizing history and the literature on social integration and mentorship, we followed 100 Native science and engineering scholars across a year of participation in the hybrid American Indian Science and Engineering Society mentorship program. The results showed that high-quality faculty mentorship predicted persistence a year later. Furthermore, mentors who shared knowledge of Native culture—through experience or shared heritage—uniquely contributed to the Native scholars’ social integration and persistence through scientific community values in particular. Therefore, Native scholars may benefit from mentorship supporting the integration of their Native culture and discipline rather than assimilation into the dominant disciplinary culture.

Native people (Native American, Alaskan Native,  Native Hawaiian) make up nearly 2% of the US population, but only make up about 0.01% of enrolled undergraduate students and 0.4% of the baccalaureate degrees in science, technology, engineering, and mathematics (STEM; Norris et al. 2012, NSF 2017). Furthermore, although the number of science and engineering doctoral degrees awarded to Natives rose from 103 in 2010 to 143 in 2014, this trend has not translated into an increase in Native higher education faculty in STEM (NSF 2017). These statistics demonstrate that even Native scholars, with high interest and an academic commitment to a STEM degree, are not integrating into their professional academic communities and are, instead, choosing to leave their academic careers. These statistics describe a persistent gap in diversifying the STEM workforce. However, attrition data fails to describe what supports Native scholar persistence and the assets Native scholars’ cultures provide not only to their persistence in STEM but to their STEM field in general.

## Native cultural assets influencing mentorship

Although Native scholars represent diverse cultures and histories, they share experiences of colonization and forced assimilation in the United States, resulting in the loss of their ancestral knowledge, language, and cultural identity (Mitchell 2018). Recognizing this history, recent scholarship provides some insight into how to support Native STEM workforce development (Page-Reeves et al. 2019a, Chow-Garcia et al. 2022). At this time, most research regarding Native STEM students and professionals has been qualitative, involving in-depth interviews to increase understanding about what factors contribute to sustaining interest and success in their profession (Page-Reeves et al. 2019a,b). From this body of research, there has been evidence that Native scholars bring to STEM fields a unique and valuable perspective that affects the research questions they ask and the approaches they take to answering them (Page-Reeves et al. 2017, 2019b). Their contributions, which emphasizes interconnection as opposed to atomization, infuse their fields of study with greater diversity of thought and innovation. In particular, Indigenous knowledge that recognizes relationships between all living creatures and the land are critical ways of knowing as we seek to address immense and interrelated challenges such as racial equity and climate change (Kimmerer 2013). Furthermore, Ngati Awa and Ngati Porou scholar Linda Tuhiwai Smith adds that Indigenous scholars are aware that “research is not an innocent or distant academic exercise but an activity that has something at stake and that occurs in a set of political and social conditions” (Smith 2012, p. 5). Unfortunately, having a different way of knowing than dominant Western academic approaches has historically resulted in Native scholars experiencing incongruence and then being given full responsibility for reconciling these worldviews, or abandoning their Native identities, because they often navigate their degree programs isolated from their communities and from other Native people (Marker 2016). Meanwhile, Indigenous knowledge is becoming increasingly relevant to the paradigm shift needed to address the greatest challenges of our day regarding the ecological demise of the planet—the knowledge that everything is interconnected (Capra 1996, UNESCO 2022).

Antonie Dvorakova's recent work, which used a qualitative interdisciplinary approach that included 40 Native American academics from 28 US universities who have persevered, concluded that Native scholars can transcend typical identity conflict (which requires choosing between their Native identity and their professional identity) and instead facilitate a meaningful integration of the existing incongruences resulting in resilient subjective experiences (Dvorakova 2018, 2019). Similarly, Page-Reeves and colleagues documented how Native professional STEM scholars describe arriving at a “new state” that is “unique without erasing previous personal and cultural identity frameworks that continue to be relevant in their lives” (Page-Reeves et al. 2019b, p. 189). These results suggest that it is not the abandonment of Native identity but the integration of it into the scholars’ approach to research that strengthens their work and their careers. Native scholars describe living at the intersection of two sociocultural contexts (professional and personal; Dvorakova 2019). Nizhoni Chow-Garcia similarly showed that learners reported that their Native cultural identity was related to their motivation to persist (Chow-Garcia 2016). Combined, the research suggests that mentors in STEM who are respectful and knowledgeable of Native culture may be important to Native scholar integration into their STEM career pathways.

## Integrating into STEM professional community

What does it mean for any student to integrate into a STEM career pathway? Building on Herb Kelman's classic social influences theory (Kelman 1958, 2006), longitudinal research on the Tripartite Integration Model of Social Influence (TIMSI) (Estrada et al. 2011, 2018, Hernandez et al. 2020) shows that three scientific orientations—efficacy (confidence in their ability to perform discipline related tasks and skills), identity (seeing oneself as belonging to the STEM community), and values (endorsement of the STEM community's core values)—predict intentions to pursue a science career 1 year later (Estrada et al. 2011) and actual STEM career choice 4 years after attaining an undergraduate STEM degree among persons excluded because of ethnicity and race (PEERs) who were mostly African American and Hispanic or Latino (Estrada et al. 2018). Mentors (i.e., more experienced persons who form working alliances with less experience persons [mentees] to support their personal and professional growth (National Academices of Sciences Engineering and Medicine 2019) are key social influencers. Research findings show that mentors draw mentees into STEM careers and community by providing meaningful instrumental, psychosocial, and networking support—the defining characteristics of high-quality mentoring relationships (Aikens et al. 2017, Hernandez et al. 2020, National Academies of Sciences, Engineering, and Medicine 2019, Pfund et al. 2016). There is also growing evidence that the quality of mentorship support is related to STEM students’ increased belonging, professional or disciplinary identity development, and overall confidence as scientists (Estrada et al. 2018), and that persistence is mediated through science identity and scientific community values for students, suggesting that quality mentoring experiences increase mentees’ sense of inclusion and integration into their disciplines (Estrada et al. 2018, Hernandez et al. 2018, 2020).

Within the mentorship literature, there is also evidence that perceived similarity; for example, sharing values, beliefs, and attitudes and an outlook between mentors and mentee contributes to higher-quality mentoring relationships (Ensher and Murphy 1997, Eby et al. 2013). In studies with African American or Hispanic undergraduates in STEM, similarity of values was found to be associated with quality mentorship and persistence in the field more than demographic similarity (Hernandez et al. 2017, Pedersen et al. 2022). However, similarity does not always translate to academic outcomes (Robinson et al. 2019), even though it does appear to affect mentor and mentee relations. Furthermore, research shows that when mentors provide support in areas that matter to the mentee, students are more likely to be successful in STEM (Baker and Griffin 2010, National Academies of Sciences, Engineering, and Medicine 2019). The research suggests that similarities regarding cultural understanding and knowledge could contribute to the sense of fit that is characteristic of positively impactful mentorship.

The mentorship research has focused on majority White or PEER scholars who are mostly African American or Hispanic undergraduate students. A notable exception is a recent study of American Indian STEM graduate students (Brazill et al. 2021), which showed that mentors’ cultural support was associated with increases in the students’ academic self-efficacy. Kirkness, who identifies as First Nation Cree of Manitoba, and Barnhardt, an advocate for Native Alaskan education, wrote about how Native students benefit from academic institutions that convey respect, relevance, reciprocity, and responsibility (Kirkness and Barnhardt 1991). Cultural principles of the Penobscot Nation, such as *N'dilnabamuk* (that all are in a relationship), *mambezu* (beliefs regarding having enough), *wolihkomawiw* (inner harmony), and *wikuwaculal* (the love of learning), which all emphasize interconnection, may affect what Native scholars perceive as supportive, in ways that are unique from the dominant culture's quality mentorship approach (Mitchell 2018).

## Study approach

**Figure 1.**
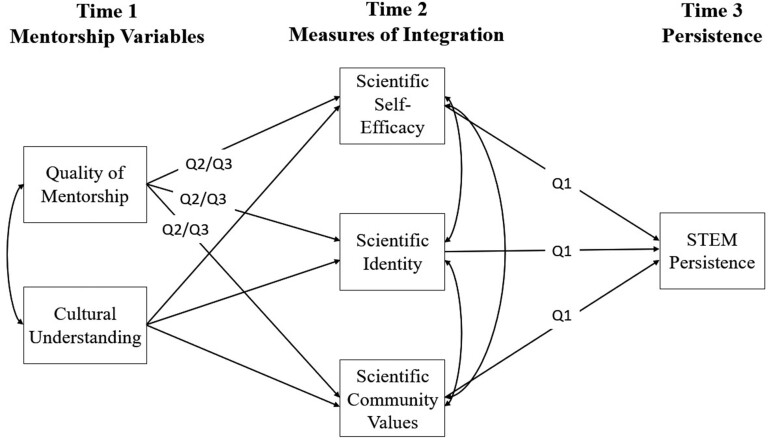
Conceptual model being tested in the current study. The paths labeled with Q1 denote paths that are related to answering our first research question. The paths labeled with Q2 or Q3 denote those related to our second and third research questions. See the text for a description of each research question. Each time point reflects 6 months after the prior time point.

Informed by the literature of social integration, mentorship, and Native culture, in this study, we seek to extend the previous research on mentorship to include a national quantitative study of Native scholars enrolled in a STEM higher education degree program, by answering three research questions. First, do measures of scientific integration—efficacy, identity, and values—predict the persistence of Native scholars in STEM fields as has been shown in previous research with African American, Latino, White, and Asian undergraduate scholars (figure 1, paths labeled as Q1)? Second, how does quality mentorship contribute to greater longitudinal social integration of Native scholars into their professional communities and persistence 12 months later (figure 1, paths labeled as Q2)? And third, does quality mentorship have unique impacts separate from mentorship that conveys shared experience or knowledge of Native culture (figure 1, paths labeled as Q3, which denote effects when taking the latter into account)?

### Participants and procedures

The participants were 100 Native American students from across the United States who were STEM majors (in science, 69%; engineering, 16%; math, technology, or interdisciplinary, 15%) and were all members of the AISES (American Indian Science and Engineering Society) Lighting the Pathway program. This program was established with the goal of increasing the number of Native faculty in STEM fields. Scholars were added each year starting in 2014 for four consecutive years (i.e., four cohorts). Baseline variables were collected the fall they were accepted into the program before starting the program and being assigned an AISES faculty mentor. The participants were surveyed every 6 months for 2 years (the response rate was 86% to 100%). Each survey included the mentorship measures (quality and cultural knowledge), measures of integration (scientific self-efficacy, identity, and community values), and STEM persistence. Time 1 (T1) in this study is defined as the first semester in which the students had an AISES assigned faculty mentor, usually by 6 months into the program. Time 2 (T2) is 6 months after T1, and time 3 (T3) is 12 months after T1. The participants were initially surveyed for the 2 years they were officially in the program, expected to participate actively in the program, and received program benefits. Although some scholars were surveyed beyond the 2 years, we will not include that data in the present article, because response attrition increased after the 2-year mark.

The participants were affiliated with 51 different tribes across 33 states, with most residing in Arizona (*n =* 9), California (*n =* 8), New Mexico (*n =* 7), and Washington (*n =* 7). Their ages ranged from 18 to 51 at the beginning of the program (*M =* 28), 63.3% were female, 35.6% were male, and 1% replied *other*. Most of the scholars (93.1%) were enrolled as students when the study began; of those enrolled, 1% were college freshmen, 11.9% were sophomores, 8.9% were juniors, 14.9% were seniors, 15.8% were master’s students, 38.9% were doctoral students, and 7.9% did not respond. Furthermore, 1% were faculty nontenure track. All scholars had the same opportunities to be paired with and interact with their mentors during their time in the 2-year program. Furthermore, all scholars were assigned an AISES-involved faculty mentor at the beginning of the program and were offered travel funding twice a year to attend the AISES National Conference and the AISES Leadership Summit to potentially interact with their mentors. They were also given access to their mentors’ contact information (e.g., email) and were encouraged to interact with their mentors as appropriate.

### Measurement of variables

To assess the mentorship variables, the quality of mentorship was measured with 10 items (*α* = .93) used in past research (Dreher and Ash 1990, Hernandez et al. 2018). This scale is composed of four items assessing psychosocial support (e.g., “To what extent has your mentor encouraged you to talk openly about anxieties and fears?”) and six items assessing instrumental support (e.g., “To what extent has your mentor helped you improve your writing skills?”). The participants responded to each item using a 1 (*not at all*) to 5 (*a very large extent*) scale.

Native cultural understanding was measured with two items (*α* = .96) referring to mentorship experiences as it related to their Native culture: “To what extent does your mentor share your cultural values?” and “To what extent does your mentor understand your cultural values?” The items were rated on a 1 (*not at all*) to 5 (*a very large extent*) scale.

The questions on cultural understanding were asked following the open-ended question “Describe cultural values that matter to you that may be different from dominant American cultural values?” Three researchers content coded the responses for common Native cultural themes adapted from the work of Kirkness and Barnhardt (1991), including respect, relevance, reciprocity, and responsibility. All 52 written responses included reference to one of these concepts, which emphasized their relationship and connection (see the supplemental material for a description of the full analysis).

To measure integration, the six-item Science Self-Efficacy Scale (Estrada et al. 2011) (*α* = .89) was used to measure the participants’ confidence in being able to perform research-related tasks. The participants were asked to rate from 1 (*not at all confident*) to 5 (*absolutely confident*) the extent they were confident in performing various research tasks, such as generating a research question to answer and creating explanations for the results of a study.

The five-item Science Identity Scale (Estrada et al. 2011; *α* = .83) was used to measure the extent to which the participants perceived themselves as members of their professional community. They were asked to rate their agreement on a scale from 1 (*strongly disagree*) to 5 (*strongly agree*) on items such as “I have a strong sense of belonging to the community of scientists” and “I have come to think of myself as a scientist.” For scholars from other majors (e.g., math), the word *scientist* was replaced with a relevant term (e.g., *mathematician*).

The four-item Scientific Community Values Scale (Estrada et al. 2011; *α* = .83) was used to measure the extent that the participants internalized the values of their professional community. The participants were asked to rate the extent to which each statement described them on a 1 (*not like me at all*) to 6 (*very much like me*) scale. The sample items included “A person who feels discovering something new in a STEM field is thrilling” and “A person who thinks it is valuable to conduct research that builds the world’s knowledge in STEM.”

The students’ intention to persist in STEM was measured with five items (*α* = .92) used in past research (Estrada et al. 2011, 2019). The participants reported their intentions for each item on a 0 (*definitely will not*) to 10 (*definitely will*) scale. The sample items included “To what extent do you intend to pursue a STEM related career?” and “To what extent do you intend to pursue a career in which you will conduct research in a STEM field?”

**Table 1a.** Intercorrelations among the variables examined in the current study.

	Mentorship Variables		Measures of Integration			Persistence
Correlation matrix	Quality of mentorship (T1)	Cultural understanding (T1)	Scientific self-efficacy (T2)	Scientific identity (T2)	Scientific community values (T2)	STEM persistence (T3)
Quality of mentorship (T1)	–					
Cultural understanding (T1)	.68***	–				
Scientific self-efficacy (T2)	.09	.07	–			
Scientific identity (T2)	.20	.37***	.45***	–		
Scientific community values (T2)	.23*	.41**	.29**	.48***	–	
STEM persistence (T3)	.27*	.34**	.17	.17	.44***	–

*Note:* T1 refers to the first semester in which the participants already had an AISES assigned faculty mentor, T2 is six months following T1, T3 is 12 months following T1. ^*^p < .05. ^**^p < .01. ^***^p < .001.

**Table 1b.** Descriptive statistics among the variables examined in the current study.

	Mentorship Variables		Measures of Integration			Persistence
Subscale descriptive statistics	Quality of mentorship (T1)	Cultural understanding (T1)	Scientific self-efficacy (T2)	Scientific identity (T2)	Scientific community values (T2)	STEM persistence (T3)
*N*	90	92	88	89	88	70
Mean	2.68	3.54	3.77	5.62	5.14	8.56
Standard deviation	1.08	1.37	0.75	1.03	.73	1.55
Cronbach's alpha	.93	.96	.89	.83	.83	.92

*Note:* T1 refers to the first semester in which the participants already had an AISES assigned faculty mentor, T2 is six months following T1, T3 is 12 months following T1. ^*^p < .05. ^**^p < .01. ^***^p < .001.

### Data analytic plan, model fit, and statistical assumptions

The degree to which our conceptual model fit the present data was examined in a structural equation modeling framework in R using maximum-likelihood estimation and the lavaan package.

We used the following fit indices to evaluate model fit: chi-square goodness of fit test (*χ*^2^), the comparative fit index (CFI; i.e., an incremental index), and the standardized root-mean-square residual (SRMR; i.e., an absolute fit index). For the chi-square goodness of fit test, a good model fit was indicated by a nonsignificant *χ*^2^ (Barrett 2007), CFI values of .95 or higher suggested a good fit (Hu and Bentler 1999), and for SRMR, a very good fit was indicated by values less than .05, and a reasonable fit was indicated by values between .05 and .10 (Browne and Cudeck 1992, Hu and Bentler 1999).

We evaluated the statistical assumptions of our structural equation model. First, the response rates varied slightly across time (see tables 1a and 1b). To determine whether the missing data were missing completely at random (MCAR; Enders 2010, 2011), Little’s MCAR test (Little 1988) was conducted. This test indicated that the pattern of missing data was consistent with MCAR, *χ*^2^(46) = 47.99, *p* = .39. Therefore, we used maximum-likelihood estimation without adjustments for missing data. Second, we screened for potential outliers by examining leverage values, studentized deleted residuals, and Cook’s *D* (Judd et al. 2009). Two outliers were detected; however, a sensitivity analysis excluding these cases did not materially affect the model fit statistics or parameter estimates, so all of the reported results include these two cases. Third, residual diagnostics showed that the linearity, normality of residuals, and homoscedasticity assumptions were met. All of our models controlled for the scholars’ reported scientific self-efficacy, identity, community values, and STEM persistence intentions at the start of the program. All of the data used for this study will be made available through an online portal when the parent research project is fully complete.

## Study findings

In our preliminary analysis, prior to examining the fit of our conceptual model, we inspected the intercorrelations among the mentorship, social integration, and persistence variables (table 1a). The quality of mentorship and cultural understanding at T1 were substantially positively correlated with each other, and they were both moderately positively correlated with STEM persistence intentions at T3; however, the magnitude of the associations between two mentorship variables and the social integration measures (i.e., efficacy, identity, and values) at T2 were noticeably different. Although the correlations between the quality of mentorship and the social integration measures at T2 were positive and small (*r* = .20–.27), the associations between the mentors’ cultural understanding and the social integration measures at T2 were positive and medium size (*r *= .34–.41). Furthermore, the correlations revealed that, of the social integration measures at T2, only the scientific community values exhibited a moderate, positive, and significant correlation with STEM persistence intentions at T3.

**Figure 2.**
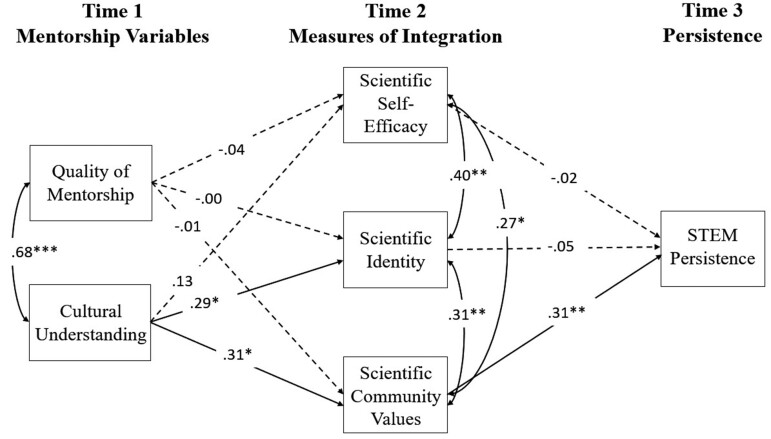
Conceptual model with parameter estimates. The dashed lines indicate nonsignificant paths. The scholars’ scientific self-efficacy, identity, values, and STEM persistence intentions reported at the start of program were controlled for but are not shown in the present figure for the sake of parsimony. Each time point reflects 6 months after the prior time point.*** ***^*^p < .05. ^*^p < .01. ^***^p < .001.

To test the longitudinal model, we estimated the data–model fit of our conceptual model, which indicated a good fit (*χ*^2^(14) = 18.27, *p* = .20, CFI = .98, and SRMR = .06). Consistent with the pattern of correlations described above, only cultural understanding at T1 had unique and significant positive correlations with scientific identity and scientific community values at T2 (figure 2). Furthermore, only scientific community values at T2 had unique and significant positive correlations on STEM persistence at T3 (figure 2). Importantly, the relationships reported above controlled for the participants’ baseline (T1) levels of scientific self-efficacy, identity, community values, and STEM persistence intentions. Taken together, these findings indicate that greater cultural understanding from mentors predicted that the Native scholars’ would have greater scientific identity and would more fully> internalize scientific community values. Furthermore, scientific community values, but not identity, predicted greater STEM persistence.

To test the longitudinal influence of the mentors’ cultural understanding at T1 on STEM persistence intentions at T3 through the social integration measures, we conducted a formal mediation analysis using a bootstrapping procedure with 10,000 iterations to construct bias-corrected 95% confidence intervals (CI) around the indirect effects (Hayes 2009). Consistent with the pattern reported above, the results showed that the mentors’ cultural understanding exerted a positive indirect influence on STEM persistence through scientific community values (indirect effect, *r* = .10, bootstrapped 95% CI = 0.02–0.26; see supplemental table S3 for the results of all mediation models tested). More specifically, the standardized indirect effect indicated that STEM persistence intentions increased by 0.10 standard deviations for every 1 standard deviation increase in the mentors’ cultural understanding through scientific community values.

## Impacts of mentorship for scholars

Following 100 Native STEM scholars across a year of participation in the AISES hybrid online and in-person mentorship program, we were able to learn what type of mentorship contributes to persistence. Similar to studies with African American and Latino STEM scholars, our Native scholars’ data showed that quality mentorship, which provides psychosocial, instrumental, and networking support, correlated with persistence a year later. However, providing quality mentorship did not uniquely predict that the scholars would integrate into their professional communities—measured by increased scientific self-efficacy, identity, or values. Instead, the results showed that the mentors’ understanding of Native culture—through experience or shared heritage—uniquely contributed to the Native scholars’ ultimate persistence. Furthermore, the results show that the Native scholars with mentors who had a knowledge of Native culture were more likely to describe themselves as someone who identifies as a scientist and internalizes the values of the scientific community (e.g., scientific research can solve many of today's world challenges). The data shows that STEM mentors who have knowledge of Native cultural ways are particularly important for Native scholars’ integration and that those Native scholars who internalize these scientific values are more likely to persist.

In some ways, these findings may seem counterintuitive if one's goal is to assimilate people into the academic discipline culture, as has been the tradition. Why would having a mentor show understanding of Native cultural values be more able to promote Native scholars’ integration into their professional careers than one who provides high-quality mentorship support (i.e., psychosocial, instrumental, and networking support) alone? One answer is that these culturally knowledgeable mentors are more similar to the scholars and provide a better fit. Previous research with dominant-culture students would predict that a mentor that fits contributes to greater mentee success. An additional explanation is that mentors with Native cultural knowledge more effectively contribute to Native scholars’ integration into their STEM career pathways because they can mentor a way forward that uses both Native and Western knowledge. Previous research, involving 40 ethnographic interviews of Native STEM scholars, suggests that Native scholars engage in a *wayfinding* process in which they find “a new way of being in, engaging with, and experiencing the world” that allows for Native knowledge to contribute with Western STEM knowledge as they navigate their professional careers (Page-Reeves et al. 2019b>, p. 189). Having mentors who are able to provide guidance on how this is achieved because of shared knowledge, acquired through shared life experiences or learning, may be particularly important for Native scholars who share a common experience of erasure and a strong commitment to maintaining Native ways of knowing and being, even in predominantly White academic environments. As is suggested by our results, mentors who can model or understand how there can be harmony between Native and STEM community values may be particularly critical to increasing Native scholar retention.

The study results do contain caveats. The participants in this study ranged from undergraduates to graduate students. This introduces variance into the model that would likely weaken findings that are truer for the students of a certain educational standing. Second, the relationship between professional identity and persistence was not significant in this study but has been shown to be very predictive in previous studies. This finding may indicate that, for Native students, sharing the values of the STEM community may be more important in career decision-making than identifying as a member of a STEM field. Alternatively, the outcome may be due to the participants in this study being 54% graduate students. Previous research has shown the importance of identity in predicting persistence among undergraduates (Chemers et al. 2011, Estrada et al. 2011, 2018, Henandez et al. 2020). Future research should assess whether this outcome is due to career stage or culture. Other measures of value alignment may also advance the research in this area. Also of importance is to acknowledge that this study was of a descriptive nature, with no control group. Therefore, future research is needed to determine whether the importance of a STEM mentor having cultural understanding is unique or more beneficial to the Native American student population than other ethnic groups. A final caveat is that the participants in this study come from over 51 tribes, each of which has its own traditions, cultures, and nuances. Future research may want to consider comparing groups through regions or common traditions.

In developing this study, Native scholars were provided the opportunity to identify their needs related to persistence in STEM and to reflect on the factors contributing to their persistence. The program, as well as the research, made a significant departure from identifying barriers and challenges (i.e., deficits) and was instead focused on what contributes to Native scholars persisting in STEM. The quantitative findings of this study are consistent with qualitative study findings, on the basis of interviews and focus groups, that Native scholars are supported in their academic careers by mentors who understand their STEM disciplines and Native cultures. The findings suggest that programs supportive of Indigenous cultures and knowledge will be more instrumental in increasing the persistence of Native scholars in STEM. Furthermore, Native scholars that receive mentoring from people who have knowledge of both their Native and STEM disciple communities, regardless of the mentor's ethnicity, may be better able to bridge the divide of these ways of knowing and positively contribute to both Native communities and STEM disciplines.

## Supplementary Material

biac064_Supplemental_File

## Author Biographical

Mica Estrada (mica.estrada@ucsf.edu) is an associate professor, Lilibeth A. Flores (Mixteca) is a research associate, and K. Kanoho Hosoda (Kanaka Maoli) is a postdoctoral research associate in the Department of Social and Behavioral Sciences and with the Institute for Health and Aging at the University of California, San Francisco, in San Francisco, California, in the United States. Gerald Young is a doctoral student in the Social/Personality Psychology program at the University of California, Berkeley, in Berkeley, California, in the United States. Paul R. Hernandez is an associate professor in the Department of Teaching, Learning, and Culture in the College of Education and Human Development at Texas A&M University, in College Station, Texas, in the United States. Kathy DeerInWater (Citizen of Cherokee Nation of Oklahoma) is the chief program officer for the American Indian Science and Engineering Society, in Albuquerque, New Mexico, in the United States.
