# Supplementary material for: Culture and Quality Matter in Building Effective Mentorship Relationships with Native STEM Scholars
Source: Bioscience. 2022 Aug 3;72(10):999–1006. doi: 10.1093/biosci/biac064 (PMC9525125; doi:10.1093/biosci/biac064)
Supplement: biac064_Supplemental_File [file biac064_supplemental_file.docx]

**Supplementary Materials for Culture and Quality Matter in Building Effective Mentorship Relationships with Native STEM Scholars**

**Content Coding of Open-ended Description of Native Culture**

**Method**

While Native culture was not a variable used to answer the posed research questions, the open-ended question “Describe cultural values that matter to you, that may be different from dominant American cultural values?” was asked prior to the questions on cultural understanding. Fifty-two responses were written, and three researchers content coded the responses for common Native cultural themes adapted from the work of Kirkness and Barnhardt (1991), including: Respect, Relevance, Reciprocity, and Responsibility (Table S1). All researchers were of Native ancestry and familiar with Native culture. When two or more researchers coded the theme as present, the response received a 1 for that theme. When only one or no researchers coded the theme as present, the response was given a 0. In addition, word frequency counts were run with the omission of the words “culture,” “America,” and “values,” which were all terms included in the question.

**Table S1.**

Content coding thematic definitions and results

| *Concept Definitions* | *# of Respondents* | *% of Respondents* |
| --- | --- | --- |
| **Respect**: To what extent do they mention respect as a value or attribute of Native/Indigenous culture? | 21 | 38.9 |
| **Relevance**: To what extent do they mention that Native/Indigenous ways of knowing and/or community are relevant to social institutions (e.g., education, family, tribal nation(s), profession, government, the legal system, etc.) | 28 | 51.9 |
| **Reciprocity**: To what extent do they mention reciprocal relationships (give and take) as a value or attribute of Native/Indigenous culture (this can include relationships between people, the natural environment, generations, etc.)? | 28 | 51.9 |
| **Responsibility**: To what extent do they mention responsibility (to self, family, community/nation) as a value or attribute of Native/Indigenous culture (e.g., helping, giving and/or doing for others)? | 28 | 51.9 |

**Results**

Results showed that all responses included at least one of the themes. Further, relevance, reciprocity and responsibility were included in over 50% of the responses (see Table S1). A word cloud representing the responses can be seen in Figure 1, with “family” and “people” being the most frequently used words (Table S2).

Overall, the coded themes and the frequently used words all emphasized connection, interweaving, and relationship. This is consistent with previous research showing that Native or Indigenous cultures places emphasis on how everything interconnects and interweaves, as opposed to separation and independence (Kirkness & Barnhardt 1991, Mitchell 2018). When participants were later asked to rate the extent to which their mentors understood Native culture, we infer that they are referring to this conception of Native culture that was written earlier in the survey.


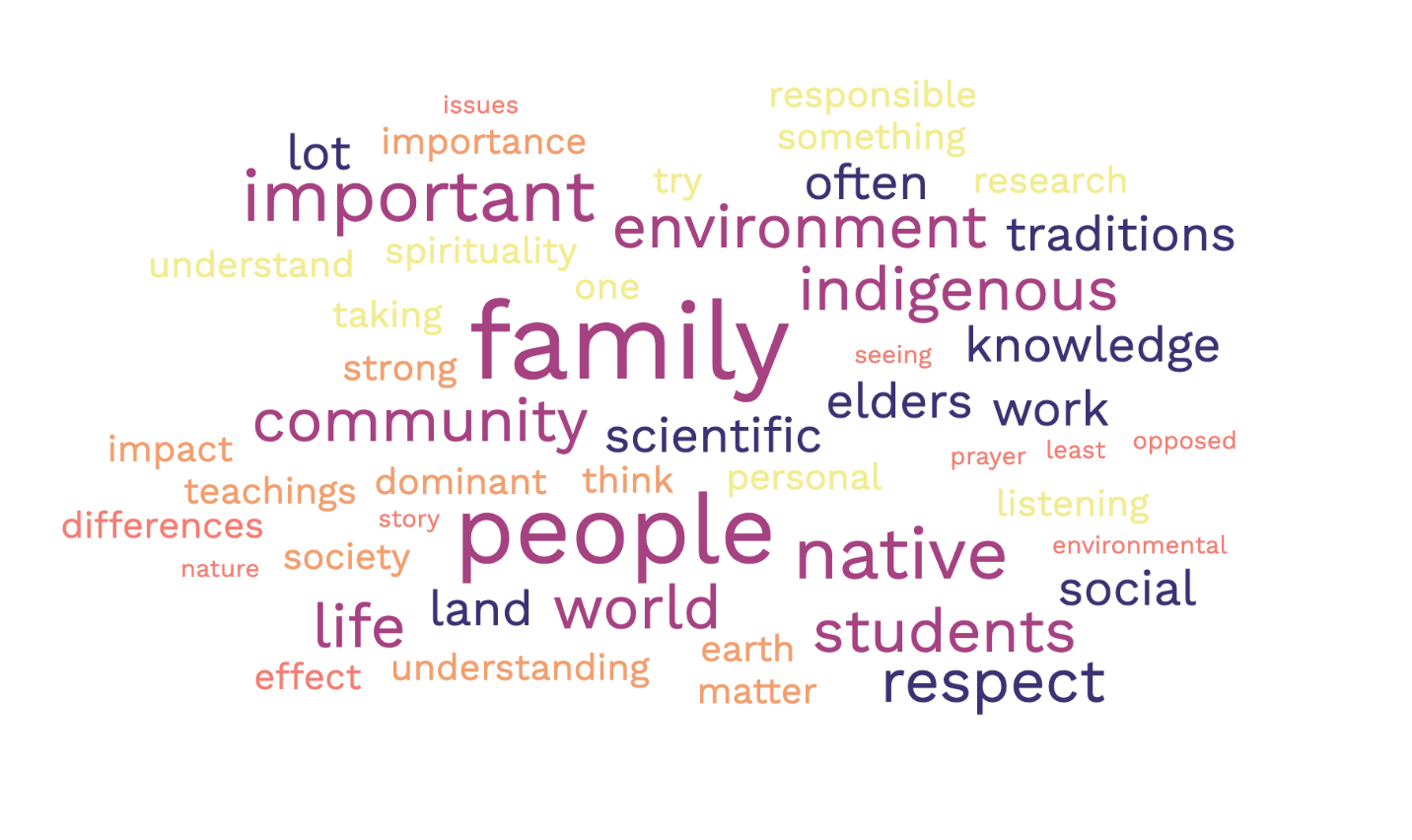
**Figure S1:** Native culture word cloud

**Table S2.** Words most mentioned and the frequency mentioned

| Word | Frequency |
| --- | --- |
| Family | 9 |
| People | 8 |
| Native | 6 |
| Important | 6 |
| World | 5 |
| Indigenous | 5 |
| Community | 5 |
| Students | 5 |
| Life | 5 |
| Environment | 5 |
| Respect | 5 |
| Scientific | 4 |

**Table S3.**

Results of the measures of integration mediating the effects of the mentorship variables on students’ STEM persistence intentions

|  | **Outcome: STEM Persistence** | | | | | | | | |
| --- | --- | --- | --- | --- | --- | --- | --- | --- | --- |
|  | a | | |  | b | | |  | a*b |
| **Mentorship Variable (x)**  Measures of Integration (m) | b | β | b/*SE* |  | b | β | b/*SE* |  | BC CI_95%_  [LL, UL] |
| **Quality of Mentorship** |  |  |  |  |  |  |  |  |  |
| Scientific Self-Efficacy | -.03 | -.04 | -.32 |  | -.08 | -.05 | -.48 |  | [-.04, .07] |
| Scientific Identity | -.00 | -.00 | -.03 |  | -.04 | -.02 | -.18 |  | [-.04, .05] |
| Scientific Community Values | -.01 | -.01 | -.09 |  | .63 | .31 | 2.64** |  | [-.11, .09] |
| **Cultural Understanding** |  |  |  |  |  |  |  |  |  |
| Scientific Self-Efficacy | .07 | .13 | .98 |  | -.08 | -.05 | -.48 |  | [-.08, .04] |
| Scientific Identity | .22 | .29 | 2.38 |  | -.04 | -.02 | -.18 |  | [-.13, .05] |
| Scientific Community Values | .17 | .31 | 2.47 |  | .63 | .31 | 2.64** |  | [.02, .26] |

*Note*. Estimates were with 10,000 bootstrap replications; b = unstandardized estimate, β = standardized estimate, b/*SE* = Z test for the statistical significance of the unstandardized estimate; BC CI_95%_ = bootstrapped bias-corrected 95% confidence intervals for the indirect effect; LL = lower limit of the confidence interval & UL = upper limit of the confidence interval. * *p* < .05, ** *p* < .01
